# The efficacy and safety of metformin combined with simvastatin in the treatment of polycystic ovary syndrome: A meta-analysis and systematic review

**DOI:** 10.1097/MD.0000000000026622

**Published:** 2021-08-06

**Authors:** Yanbo Liu, Yupei Shao, Jiping Xie, Linlin Chen, Guang Zhu

**Affiliations:** Department of Gynecology, Litongde Hospital of Zhejiang Province, China.

**Keywords:** meta-analysis, metformin, polycystic ovary syndrome, review, simvastatin

## Abstract

**Background:**

Several previous randomized controlled trials (RCTs) evaluated the efficacy of metformin combined with simvastatin in the treatment of polycystic ovary syndrome (PCOS), yet the results of the researches are not consistent. It is necessary to conduct a meta-analysis to explore the efficacy and safety of metformin combined with simvastatin in the treatment of PCOS, to provide evidence supports for the treatment of PCOS.

**Methods:**

We searched PubMed, EMbase, Cochrane Library, China National Knowledge Infrastructure, Wanfang, and Chinese biomedical literature databases online to identify the RCTs evaluating the efficacy of metformin combined with simvastatin in the treatment of PCOS. Standardized mean difference (SMD) and 95% confidence interval (95% CI) were calculated to evaluate the synthesized effects.

**Results:**

Nine RCTs with a total of 746 PCOS patients were included. The synthesized results indicated that the combined use of metformin and simvastatin are more beneficial to reduce the total cholesterol (SMD –2.66, 95% CI –3.65 to –1.66), triglycerides (SMD –1.25, 95% CI –2.02 to –0.49), low density lipoprotein (SMD –2.91, 95% CI –3.98 to –1.84), testosterone (SMD –0.64, 95% CI –1.13 to –0.15), fasting insulin (SMD –1.17, 95% CI –2.09 to –0.26) than metformin alone treatment in PCOS patients (all *P* < .001), and there was no significant difference in the high density lipoprotein (SMD –0.05, 95% CI –0.56–0.46), luteinizing hormone (SMD –0.58, 95% CI –1.66 to –0.50), follicle stimulating hormone (SMD 0.41, 95% CI –0.78–1.59), prolactin (SMD –1.38, 95% CI –2.93–0.17), fasting blood sugar (SMD 0.23, 95% CI –0.52–0.97), and insulin sensitivity index (SMD –0.17, 95% CI –0.48–0.15) between experimental and control groups (all *P* > .05).

**Conclusions:**

Metformin combined with simvastatin is superior to metformin alone in the treatment of PCOS patients with more advantages in improving the levels of sex hormones, blood lipids, and blood sugar. However, the safety of this therapy still needs to be further explored in clinical studies with high-quality and large samples.

## 1 Introduction

Polycystic ovary syndrome (PCOS) is the most common endocrine disease in women of childbearing age.^[1]^ It is characterized by chronic ovulation dysfunction and hyperandrogenemia, affecting 3.84% to 10.11% of women of childbearing age worldwide.^[2,3]^ PCOS is often accompanied by risks of abdominal obesity, insulin resistance, metabolic disorders, and cardiovascular disease.^[4,5]^ Metformin is one of the most commonly used drugs in the treatment of PCOS at present. It can participate in the metabolism of liver, muscle, and adipose tissue, effectively reducing the clinical symptoms of insulin resistance and excessive androgen expression.^[6]^ The main adverse reaction is intestinal symptoms, including nausea, bloating, indigestion, diarrhea, flatulence, metallic taste, and even anorexia.^[7,8]^ Most of the symptoms are relieved within 1 to 2 weeks of continuous use of the drug, and only a few patients have lactic acid.^[9]^ Therefore, the prevention and treatment of metformin-related complications are of great significance for the prognosis of patients with PCOS.

Metformin has the effect of enhancing insulin sensitivity, regulating lipid metabolism, and inflammation, and this effect has been widely used in many fields.^[10]^ In the past few decades, women with gestational diabetes, PCOS, and obesity have gradually received metformin treatment.^[11]^ Taking statins has been used for treating PCOS in recent years.^[12]^ Statins can reduce the morbidity and mortality of cardiovascular diseases, reduce the level of androgen in serum, improve blood lipid profile and endothelial function.^[13]^ And at the same time, it has a positive protective effect on cardiovascular function as an anti-inflammatory and antioxidant.^[14]^ Metformin combined with simvastatin is theoretically beneficial to the treatment of PCOS, and it can improve blood sugar, blood lipids, and sex hormone levels.^[15]^ However, there is still a lack of reliable evidences of multicenter large sample data to prove the superiority of combined use of metformin and simvastatin in the treatment of PCOS.^[16]^ Therefore, it's necessary to explore whether the combination of metformin and simvastatin has advantages over metformin alone in the treatment of PCOS, we aimed to conduct a meta-analysis and systematic review to evaluate the effects and safety of combined use of metformin and simvastatin in the treatment of PCOS, to provide medical evidences for the treatment of PCOS.

## 2 Methods

We tried to conduct and report this meta-analysis in comply with the Preferred Reporting Items for Systematic Reviews and Meta-Analyses (PRISMA). Ethical approval was not necessary since our study was a meta-analysis.

### 2.1 Search strategy

We searched PubMed, EMbase, Cochrane Library, China National Knowledge Infrastructure, Wanfang, and Chinese biomedical literature databases online. The search time is limited from the establishment of the database to November 30, 2020. The search terms used included: polycystic ovarian syndrome, polycystic ovary syndrome, PCOS, metfomin, dimethyl biguanide, SimVastatin, randomized controlled trial, RCT. We used the above search terms as subject terms or free words, and used logical operators to formulate corresponding search formulas. Relevant references of the related reviews were searched manually. And we tried to contact the corresponding author for missing data if necessary.

### 2.2 Inclusion and exclusion criteria

The inclusion criteria of this study were: Research study design: The randomized controlled trial (RCT) of metformin combined with simvastatin in the treatment of PCOS, and the language was limited to Chinese and English. Research objects: All PCOS patients were included, and the PCOS diagnosis met the corresponding diagnostic criteria. Intervention measures: The experimental group was treated with metformin combined with simvastatin for PCOS, and the control group was treated with metformin for PCOS. Outcomes: total cholesterol (TC), triglycerides (TG), low density lipoprotein (LDL), high density lipoprotein (HDL), testosterone (T), luteinizing hormone, follicle stimulating hormone (FSH), prolactin, fasting blood sugar (FBS), fasting insulin (FIN), insulin sensitivity index. The included literature should include at least one of the above outcomes. The exclusion criteria of this study are: other diseases that cause hyperandrogenism such as congenital adrenal hyperplasia, cushing syndrome, androgen-secreting tumors, etc. Other diseases that may cause ovulation disorders such as premature ovarian failure, hyperprolactinemia, hypothalamic or pituitary amenorrhea, and abnormal thyroid function, etc. Patients taking hormones or lipid metabolism regulating drugs within 3 months. Patients during pregnancy and lactation. Patients with abnormal liver and kidney function or other serious cardiovascular diseases. Non-RCT studies.

### 2.3 Literature screening and data extraction

Two researchers independently screened the literature according to the literature inclusion and exclusion criteria, then read the selected literature, and extracted the relevant research data. If there were any further queries, they discussed the differences and let the third researcher for some determination. The basic extracted information included the first author, the year of publication, the country, the sample size, and the details of intervention measures including the dosage of metformin and simvastatin, the frequency of medication, and the course of treatment. At the same time, the corresponding observation indicators were extracted.

### 2.4 Statistical analysis

We used Review manager 5.2 software to carry out relevant statistical analysis, and the data of the experimental and control group were expressed as ¯x ± *s*. Continuous variables were described by standardized mean difference (SMD) and its 95% confidence interval (95% CI), and binary variables were described by odds ratio (OR) and its 95% CI. The heterogeneity of the included studies was evaluated by *Q* test, and the heterogeneity was expressed as *I*^2^. The random effects model was used for analysis. We used STATA 12.1 software to evaluate publication bias, with *P* < .05 means the difference was statistically significant.

## 3 Results

### 3.1 Literature search

**Figure 1:**
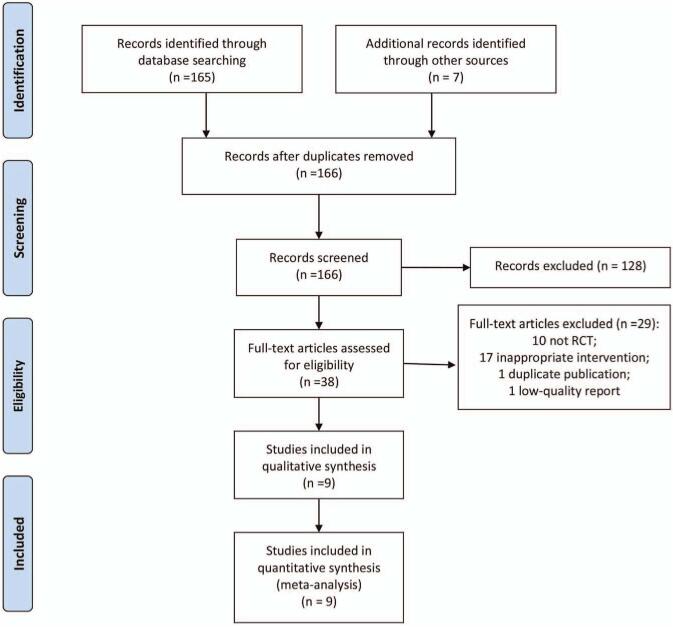
The flow diagram of study selection.

As presented in Fig. 1, at the beginning, we searched 166 articles by computer after remove the duplicate publications and inconsistent articles. After reading the title and abstract and further reading the full text, we finally included 9 RCTs.^[17–25]^

### 3.2 The characteristics of studies

As showed in Table 1, of the included 9 RCTs, a total of 746 patients were included, with 374 patients received the metformin combined with simvastatin treatment, and 372 patients underwent the metformin treatment. Four RCTs were conducted in China,^[21–24]^ 2 in Poland,^[17,19]^ 1 in Iran,^[18]^ USA,^[20]^ and Pakistan^[25]^ respectively. The dose of metformin varied from 500 to 850 mg/time, and the Simvastatin was administered 20 mg/d orally in all included RCTs. And duration of follow-up differed from 2 months to 6 months.

**Table 1:** The characteristics of included RCTs.

		Sample size		Interventions		
Studies	Countries	Experimental group (n = 374)	Control group (n = 372)	Experimental group (n = 374)	Control group (n = 372)	Duration of follow-up
Banaszewska 2009	Poland	37	36	Metformin 850 mg/time, twice a day, orally+ Simvastatin 20 mg/d orally	Metformin 850 mg/time, twice a day, orally	3 months
Kazeronni 2010	Iran	42	42	Metformin 850 mg/time, 3 times a day, orally+ Simvastatin 20 mg/d orally	Metformin 850 mg/time, 3 times a day, orally	12 weeks
Banaszewska 2011	Poland	36	33	Metformin 850 mg/time, twice a day, orally+ Simvastatin 20 mg/d orally	Metformin 850 mg/time, twice a day, orally	6 months
Karakas 2013	USA	18	20	Metformin 850 mg/time, twice a day, orally+ Simvastatin 20 mg/d orally	Metformin 850 mg/time, twice a day, orally	3 months
Shi 2013	China	23	23	Metformin 500 mg/time, twice a day, orally+ Simvastatin 20 mg/d orally	Metformin 500 mg/time, twice a day, orally	4 months
Wang 2014	China	66	66	Metformin 500 mg/time, twice a day, orally+ Simvastatin 20 mg/d orally	Metformin 500 mg/time, twice a day, orally	63 days
Xiao 2014	China	38	38	Metformin 500 mg/time,3 times a day, orally+ Simvastatin 20 mg/d orally	Metformin 500 mg/time, 3 times a day, orally	63 days
Wang 2017	China	60	60	Metformin 500 mg/time, 3 times a day, orally+ Simvastatin 20 mg/d orally	Metformin 500 mg/time, 3 times a day, orally	3 months
Malik 2018	Pakistan	54	54	Metformin 500 mg/time, 3 times a day, orally+ Simvastatin 20 mg/d orally	Metformin 500 mg/time, 3 times a day, orally	3 months

### 3.3 The quality of included RCTs

**Figure 2:**
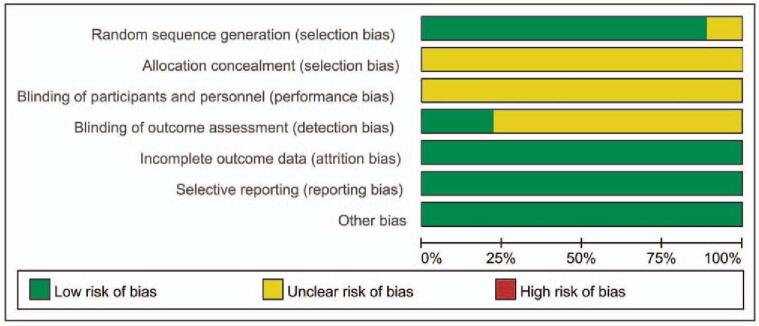
Risk of bias graph.

**Figure 3:**
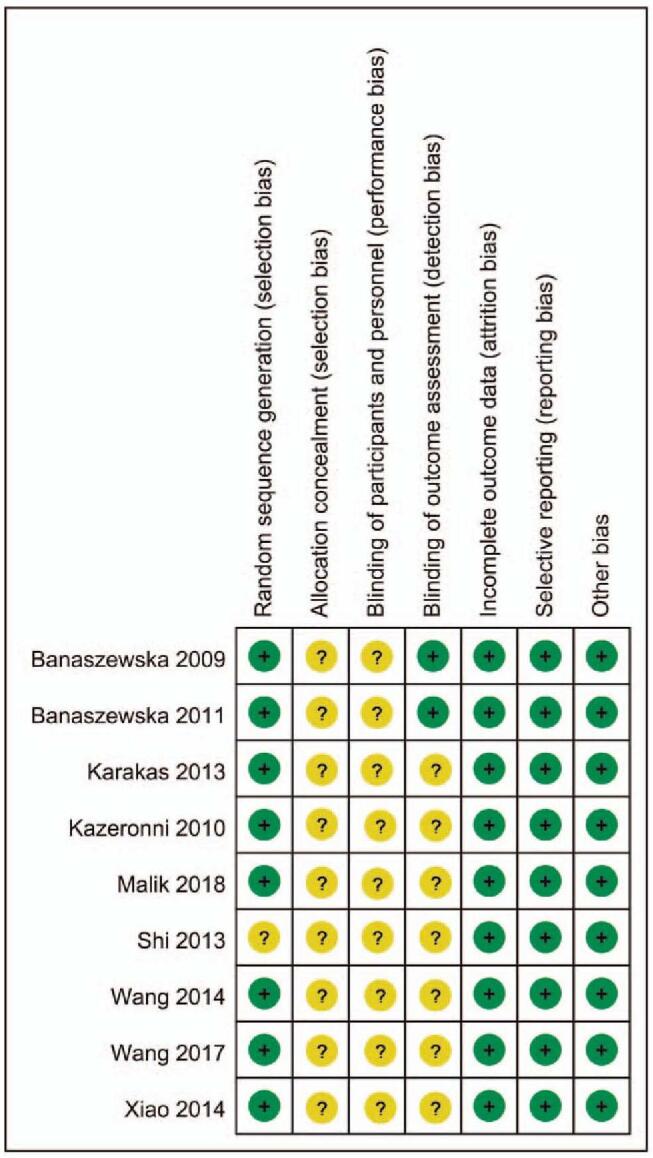
Risk of bias summary.

The quality of included RCTs was presented in Figs. 2 and 3. Even through all the included RCTs mentioned the randomized controls, and the efforts to minimize the risk of incomplete data results and selective publication bias, one RCT^[21]^ didn’t report the details of how to generate the randomized sequence. And all the included RCTs did not report the blinding design in the allocation process and participants. Only 2 studies^[17,19]^ reported the binding design in the outcome assessment, the resting did not report the blinding design. No other biases were found amongst the included RCTs.

### 3.4 Synthesized outcome analysis

#### 3.4.1 Blood lipid

As presented in Table 2, the combined use of metformin and simvastatin are more beneficial to reduce the TC (SMD –2.66, 95% CI –3.65 to –1.66), TG (SMD –1.25, 95% CI –2.02 to –0.49), LDL (SMD –2.91, 95% CI –3.98 to –1.84) than metformin alone treatment in PCOS patients (all *P* < .001), and there was no significant difference in the HDL (SMD –0.05, 95% CI –0.56–0.46) between experimental and control groups (*P* = .844).

**Table 2:** Meta-analyses on the effects of blood lipid.

		Heterogeneity		Random effects model		
Variables	Number of included RCTs	*I*^2^	*P*	SMD	95% CI	*P*
TC	8	96%	<.001	−2.66	−3.65 to −1.66	<.001
TG	8	95%	<.001	−1.25	−2.02 to −0.49	.001
LDL	8	96%	<.001	−2.91	−3.98 to −1.84	<.001
HDL	7	88%	<.001	−0.05	−0.56–0.46	.844

#### 3.4.2 Sex hormones

As presented in Table 3, the combined use of metformin and simvastatin are more beneficial to reduce the T level (SMD –0.64, 95% CI –1.13 to –0.15) than metformin alone treatment in PCOS patients (*P* = .012), and there was no significant difference in the LH (SMD –0.58, 95%CI –1.66 to –0.50), FSH (SMD 0.41, 95% CI –0.78–1.59), prolactin (SMD –1.38, 95% CI –2.93–0.17) between experimental and control groups (all *P* > 0.05).

**Table 3:** Meta-analyses on the effects of sex hormones.

		Heterogeneity		Random effects model		
Variables	Number of included RCTs	*I*^2^	*P*	SMD	95% CI	*P*
T	7	88%	<.001	−0.64	−1.13 to −0.15	.012
LH	6	97%	<.001	−0.58	−1.66 to −0.50	.291
FSH	6	97%	<.001	0.41	−0.78–1.59	.502
PRL	3	96%	<.001	−1.38	−2.93–0.17	.085

#### 3.4.3 Blood glucose

As presented in Table 4, the combined use of metformin and simvastatin are more beneficial to reduce the FIN level (SMD –1.17, 95% CI –2.09 to –0.26) than metformin alone treatment in PCOS patients (*P* = .014), and there was no significant difference in the FBS (SMD 0.23, 95% CI –0.52–0.97) and insulin sensitivity index (SMD –0.17, 95% CI –0.48–0.15) between experimental and control groups (all *P* > .05).

**Table 4:** Meta-analyses on the effects of blood glucose.

		Heterogeneity		Random effects model		
Variables	Number of included RCTs	*I*^2^	*P*	SMD	95% CI	*P*
FBS	7	93%	<.001	0.23	−0.52–0.97	.552
FIN	7	95%	<.001	−1.17	−2.09 to −0.26	.014
ISI	4	38%	.192	−0.17	−0.48–0.15	.301

No subgroup analyses were performed in our study because the interventions of included studies differed remarkably. We attempted to evaluate publication bias with a funnel plot. And the dots were scattered symmetrically and evenly, and no bias was found in the outcomes. Sensitivity analyses, which investigated the influence of one single study on the overall risk estimate by removing study one by one, suggested that the overall risk estimates were not substantially changed by any single study.

## 4 Discussion

The typical characteristics of PCOS mainly include abnormal gonadotropin ratio, anovulation, irregular menstruation, insulin resistance, dyslipidemia, vascular and endothelial dysfunction, elevated androgen levels, and polycystic ovary morphology during ultrasound imaging.^[26]^ It has been reported in the literature that the risk of type 2 diabetes, metabolic syndrome, and cardiovascular disease in patients with PCOS is significantly higher than the risk of ordinary people suffering from these diseases.^[27]^ Studies^[28,29]^ have shown that hyperandrogenemia is one of the important factors that promote the stagnant development of follicles and ultimately lead to anovulation. Therefore, the early treatment of PCOS is of great significance to the prognosis of patients. With 9 RCTs included, this present meta-analyses have found that the combined use of metformin and simvastatin are more beneficial to reduce the TC, TG, LDL, T, and FIN level when compared with metformin alone treatment in PCOS patients. Therefore, the combined use of metformin and simvastatin are more effective in the treatment of PCOS.

Metformin is a class of anti-hyperglycemic guanidine drugs used in the treatment of type 2 diabetes.^[30]^ Studies^[31,32]^ have shown that metformin reduces the concentration of blood lipids by reducing the concentration of TG, TC, and LDL in the plasma. In addition, metformin can also affect the sensitivity of insulin by increasing gluconeogenesis, thereby inhibiting hepatic glucose production.^[33]^ At the same time, it improves the sensitivity of tissues to insulin, slows down the absorption of glucose in the gastrointestinal tract, and reduces free T levels, thereby improving the symptoms of hirsutism. About 38.14% of PCOS patients can achieve a normal ovulation cycle and reduce hirsutism after metformin treatment.^[34]^ Statins are currently a novel drug for the treatment of PCOS. They act by competitively inhibiting the first stage of the mevalonate pathway, 3-hydroxy-3-methylglutaryl-coenzyme A (HMG-cOA) reductase, leading to a decrease in cholesterol synthesis and a compensatory increase in the expression of LDL receptors in the liver, and it may affect glucose homeostasis mainly by inhibiting insulin secretion in pancreatic B cells.^[35]^ Besides, Simvastatin can attenuate the signals related to insulin and insulin-like growth factors in ovarian cells, reverse the proliferation of follicular membranes and reduce the production of steroid-producing enzymes, thereby promoting ovulation.^[36]^

Studies^[27,37]^ have shown that simvastatin can effectively improve the blood lipid metabolism of PCOS patients. Then, whether the 2 drugs can be used to optimize the treatment effect, this study has explored and verified in this aspect. Studies^[38,39]^ have shown that after combined use with blood lipid metabolism regulating drugs, the insulin sensitization effect, sex hormone regulation effect of metformin, and the blood lipid regulation effect of simvastatin have been significantly improved, and more satisfactory therapeutic effects have been obtained. It is worth noting that the research in this area is still relatively inadequate. It is of very important clinical value to carry out such clinical studies as to whether the combined medication can significantly improve body mass indicators, sex hormone levels, and blood glucose and lipid metabolism. Studies^[40,41]^ have pointed out that metformin combined with simvastatin may have a better therapeutic effect than a single drug in the treatment of PCOS, but there is still a lack of sufficient medical evidence. Combined medication has a significant effect in reducing blood lipids, T and FIN in PCOS patients, and the difference is statistically significant. Simvastatin combined with metformin may have a synergistic effect in reducing patients’ blood lipids, T and FIN, but may have no effect on other indicators, such as FBS, BMI, HDL, etc. The specific mechanism of action is not clear. According to previous reports,^[42,43]^ metformin can significantly reduce FBS levels, but cannot reduce CRP levels, and metformin combined with simvastatin can reduce BMI and TG levels, but does not change HDL levels. Other studies^[36,44,45]^ have shown that simvastatin significantly reduces some biochemical parameters, such as FSH, LH, T, Tc, LDL, and increases HDL levels, while metformin significantly reduces FSH, T, and TC levels. This study further showed that the difference between metformin combined with simvastatin and metformin in reducing FSH levels is not statistically significant, but has a significant effect in reducing LDL levels, but it does not reduce luteinizing hormone levels. Studies^[27,46]^ have shown that compared with simvastatin, metformin can improve the level of FIN better. The results of this study show that the combination can significantly reduce the level of FIN in patients, and the difference is statistically significant. It may be due to the improvement of insulin resistance and enhancement of insulin sensitivity.

It is worth noting that whether the dose of metformin is 500 mg/time, 3 times a day orally, or 850 mg/time, 2 times a day, and how long the treatment is more beneficial to PCOS patients are still controversial. Statins are generally considered safe, especially for long-term use. Side effects of statins mainly include head pain, trouble sleeping, drowsiness, dizziness, cramps, nausea or vomiting, abdominal pain, bloating, diarrhea, constipation, and skin rash.^[47,48]^ Another uncommon side effect is liver toxicity, especially it is more obvious in patients with active liver disease. Therefore, the safety of metformin combined with simvastatin in the treatment of PCOs still needs more related studies in the future.

This study has certain shortcomings. First of all, the number of included RCTs was small and the quality of the RCTs was average, which needs to be further verified by future multicenter, large-sample clinical studies. Secondly, the heterogeneity of the results of this study was relatively high. The source of the heterogeneity may be mainly related to the patient's condition, course of disease, course of treatment, dosage, countries, and ethnicity. We tried to make further subgroup analyses, but we still cannot eliminate the corresponding heterogeneity. Thirdly, there are insufficient reports on the adverse reactions after medication in the included literature, and only one article reported the occurrence of gastrointestinal adverse reactions in both groups. Therefore, more relevant studies are necessary to explore the safety of combined use of metformin and simvastatin.

## 5 Conclusions

In summary, compared with metformin alone, metformin combined with simvastatin can significantly reduce the levels of TC, TG, LDL, T, and FIN in PCOS patients. However, only one RCT reported adverse reactions amongst the included 9 RCTs, more attentions are still needed to ensure the safety of combined use of metformin and simvastatin. Therefore, the efficacy and safety of metformin combined with simvastatin in the treatment of PCOS still need to be further verified by large-sample, multicenter, high-quality RCTs.

## Author contributions

**Conceptualization:** Yanbo Liu, Yupei Shao, Guang Zhu.

**Data curation:** Yupei Shao, Jiping Xie, Linlin Chen, Guang Zhu.

**Formal analysis:** Yanbo Liu, Yupei Shao, Linlin Chen, Guang Zhu.

**Investigation:** Jiping Xie, Linlin Chen, Guang Zhu.

**Methodology:** Yanbo Liu, Jiping Xie.

**Project administration:** Linlin Chen.

**Resources:** Yanbo Liu, Yupei Shao, Guang Zhu.

**Software:** Yanbo Liu, Yupei Shao, Linlin Chen.

**Supervision:** Yanbo Liu, Guang Zhu.

**Validation:** Yanbo Liu, Yupei Shao, Jiping Xie, Linlin Chen.

**Visualization:** Yanbo Liu, Yupei Shao.

**Writing – original draft:** Yupei Shao, Jiping Xie.
